# Medical News and Miscellany

**Published:** 1888-07

**Authors:** 


### MEDICAL NEWS AND MISCELLANY.

TRANSPLANTATION OF NERVE FROM A RABBIT TO MAN.—The N. Y. Record, of June 9th, contains an account taken from the Vienna correspondent of the *British Medical Journal* of a unique operation. The operator was Dr, Gersung, an assistant of Billroth, and the patient was Professor Fleischel. Prof. Fleischel received a wound sixteen years ago, while holding a post mortem examination, which resulted in inflammation of the right hand and arm, and finally, gangrene of the terminal phalanx of the thumb, which was amputated. Neuromata developed, attended with great pain, and the branches of the radial and median nerves were successively resected, until the thumb and nearly all the forefinger became anesthetic. New neuromata continued to develope, however, and the pains became so great that the patient wished to undergo another operation, in order to procure at least some temporary relief. Tho neuroma was excised and the ends of the nerves cut smooth. About two and one-third inches of the sciatic nerve of a rabbit was then inserted between the central stump of the median nerve and its digital branches and then properly sutured. Healing took place by first intention. All pain ceased soon after the operation. Two months have now elapsed, and sensibility is becoming reestablished in the parts. The ultimate result of this operation will be awaited with great interest.

---

THE SOUTHERN SURGICAL AND GYNÆCOLOGICAL ASSOCIATION will meet at Birmingham, Alabama, on the 11th of September and hold three days. We acknowledge the courtesy of a special invitation. A very profitable meeting is anticipated. Dr. W. E. B. Davis, of Birmingham, Ala., is Secretary.

---

ANTIDOTE TO SERPENT VENOM.—Dr. H. C. Yarrow, Curator of Reptiles in the National Museum (*Forest and Stream*, May 10th) announced that he has discovered the fluid extract of jaborandi to be an efficient antidote to serpent venom. This applies to mammals, but not to birds.—*N. Y. Record*, June 9.

---

"INCONTINENCE IN MALES."—In the report of proceedings of one of our leading county medical societies, our reporter says: "Dr. Simonton reported a case of incontenence in an adult male."

We are rather of the opinion that such cases are quite common, if the wives of certain of our citizens could be consulted; it is a chronic complaint amongst certain "adult male" married men; and if Dr. Simonton knows a positive cure, he could make a fortune by the judicious use of it. Of course, the reporter meant incontinence of urine; but incontinence, unless qualified, is a dangerous word.

---

BAFFLED SCIENCE.—Under this head the "*Austin Statesman*" tells of a case at the Lunatic Asylum, in which the symptoms could not be properly interpreted by the physicians, and on the death of the patient, a female lunatic, "a *post mortem* examination" was made, "after death," says the *Statesman*, and in the woman's stomach was found a ball of hair, the size of a hickory nut. It is supposed, the woman had a habit of swallowing the combings of her hair, and perhaps that of others, and it had formed into this mass. *Too* bad, the Doctors could'nt make that *post mortem before* death! We are reminded of one of the *Piofessors* in a certain medical college, who instructed the class in a certain operation, to "make a subcutaneous incision under the skin," and of a showman, who advertised "a pyrotechnic display of fireworks." It is in print that a certain instrument has "an indellible mark that won't rub off."

---

WE have received a neat little pamphlet entitled, "A Brochure on the Hypodermic Treatment of Internal Hemorrhoids by Q. A. Shuford, M. D., of Tyler, Texas," and also a communication from Dr. Shuford on "The Glycerole Compound as a remedy for Internal Hemorrhoids." The latter will appear in the next issue of the JOURNAL.

---

PLANT TREES ON YOUR FARM.—George Pinney, Evergreen, Door county, Wisconsin, will send you twenty-seven thousand sugar maple seedlings for $15, boxed and shipped: one hundred thousand arbor vitæ seedlings for $50, (which is 50c. per thousand); small lots in proportion. mention this journal.

---

YELLOW FEVER IN MISSISSIPPI.—The Marine Hospital Bureau, at Washington, is informed of the arrival at Ship Island quarantine, Mississippi, of the Norwegian bark, Magnolia, from Rio Janairo. The captain and four of the crew died from yellow fever, on the voyage. Vessel detained at quarantine.

---

MISS JESSEE PATTON, daughter of Dr. A. L. Patton, of Minneola, Texas, graduated Batchelor of Arts, and Master of Arts at the University of Texas in June. She should now add "M. D." to her name, she would donbtless make a good one; it runs in the family.

---

WE REGRET to learn of the death, recently, of Mrs. Barton, wife of Dr. W. A. Barton, of Newport.

REED & CARNRICK have issued a neat little book for the physician's pocket, consisting of a well regulated diet table for the sick. The leaves can be torn out and handed the nurse along with the prescription, each article of diet suitable to the case having been marked by the doctor. Each leaf contains also a list of what articles should be avoided. Free on application. See their advertisement for address.

---

A NEW INSTRUMENT, TEXAS INVENTION.—Dr. J. W. Daniel, of Houston, sends us a drawing and description of an invention of his own for passing close stricture of the urethra. It consists of a syringe with a screw nozzle for the attachment of catheters of different sizes. Dr. Daniel writes:

"The old plan of injecting oil into the urethra, grasping the penis tightly, to retain the fluid, and then introducing the sound, or catheter, is familiar to all. Here I present an instrument which combines the injecting apparatus with the dilating instrument. With it, the parts can be distended with fluid—warm oil, for instance—while the instrument [catheter attached, and through which the fluid has been injected,] can be made to follow the fluid as it finds its way through the strictured portion of the canal. Further description is unnecessary, as its objects and uses can be readily understood from the above. The catheter attachment is made to screw on, and may be of any size."

---

ANOTHER NEW JOURNAL.—Vol. I., No. 1, of the Medical Investigator, is before us. It is published monthly, by S. F. Smith, M. D., at Louisville, Ky. It is devoted to medicine and temperance, maintaining that the liquor traffic should be dealt with by the State and National governments as a sanitary question. It sets its face against physicians patronizing druggists who prescribe and vend patent medicines. Attention is called to the inconsistency of condemning homeopathic and other advertising quacks, when we patronize vendors of patent nostrums, and, too, in some instances, when the vendors are regular practicing physicians in good standing.

